# Establishment and Characterization of 7 Novel Hepatocellular Carcinoma Cell Lines from Patient-Derived Tumor Xenografts

**DOI:** 10.1371/journal.pone.0085308

**Published:** 2014-01-09

**Authors:** Hong Xin, Ke Wang, Gang Hu, Fubo Xie, Kedong Ouyang, Xuzhen Tang, Minjun Wang, Danyi Wen, Yizhun Zhu, Xiaoran Qin

**Affiliations:** 1 Shanghai ChemPartner Co., LTD, Shanghai, China; 2 Department of Pharmacology, School of Pharmacy, Fudan University, Shanghai, China; National Institutes of Health, United States of America

## Abstract

Hepatocellular carcinoma (HCC) is a common cancer with poor prognosis worldwide and the molecular mechanism is not well understood. This study aimed to establish a collection of human HCC cell lines from patient-derived xenograft (PDX) models. From the 20 surgical HCC sample collections, 7 tumors were successfully developed in immunodeficient mice and further established 7 novel HCC cell lines (LIXC002, LIXC003, LIXC004, LIXC006, LIXC011, LIXC012 and CPL0903) by primary culture. The characterization of cell lines was defined by morphology, growth kinetics, cell cycle, chromosome analysis, short tandem repeat (STR) analysis, molecular profile, and tumorigenicity. Additionally, response to clinical chemotherapeutics was validated both *in vitro* and *in vivo*. STR analysis indicated that all cell lines were unique cells different from known cell lines and free of contamination by bacteria or mycoplasma. The other findings were quite heterogeneous between individual lines. Chromosome aberration could be found in all cell lines. Alpha-fetoprotein was overexpressed only in 3 out of 7 cell lines. 4 cell lines expressed high level of vimentin. Ki67 was strongly stained in all cell lines. mRNA level of *retinoic acid induced protein 3 (RAI3)* was decreased in all cell lines. The 7 novel cell lines showed variable sensitivity to 8 tested compounds. LIXC011 and CPL0903 possessed multiple drug resistance property. Sorafenib inhibited xenograft tumor growth of LIXC006, but not of LIXC012. Our results indicated that the 7 novel cell lines with low passage maintaining their clinical and pathological characters could be good tools for further exploring the molecular mechanism of HCC and anti-cancer drug screening.

## Introduction

Liver cancer is one of the most common cancers worldwide. Based on a global cancer statistics study, liver cancer represents the fifth most frequently diagnosed cancer and the second most frequent cause of cancer death in men[1]. It is the seventh and sixth in women respectively. The majority of new cases and deaths come from developing countries, such as China and India. Hepatocellular carcinoma (HCC) accounts for 70% to 80% of primary liver cancers in adults[2]. Infections with hepatitis B and C virus (HBV and HCV), alcoholism and aflatoxin intake are well-defined risk factors of HCC[3]–[5]. HBV is the main cause in Africa and East Asian counties, whereas HCV is the main risk factor in the West[6]. The chronic liver inflammation associated with hepatocyte necrosis and regeneration results in cirrhosis which ultimately leads to HCC.

HCC had long been diagnosed at an advanced stage with progressive liver damage and there were no known effective therapeutic options. During the past twenty years, considerable progress has been achieved in diagnosis, therapies and life expectancy. Effective treatments include surgical resection, radio-frequency ablation, liver transplantation and percutaneous ablation[7]. The use of sorafenib in HCC patients, a multi-tyrosine kinase inhibitor, targeting membrane receptors involved in angiogenic and mitogenic intra-cellular signaling and kinases along RAS/RAF/MEK/ERK pathway, offers a new approach of HCC therapy, which is molecular targeted therapy[8], [9]. Unfortunately, most HCC patients presents with advanced or unresectable disease and, therefore, not suitable for curative therapies. High recurrence rate is another difficult challenge. HCC has high heterogeneity which leads to the extreme variability of the clinical outcome [7], [10].

Patient-derived xenograft (PDX) models have been regarded as valuable models for oncology drug development[11] and prediction of the cancer therapy[12]. In the present study, we reported the establishment and characterization of 7 new human HCC cancer cell lines from PDX models. Tumor tissues came from surgical HCC samples of Chinese HCC patients with different phenotypes. The characterization of the novel cell lines also showed the high heterogeneity of HCC. Some cell lines can be benefit to translational research and to improve preclinical optimization of personalized drug therapy.

## Materials and Methods

### Ethics Statement

This study was carried out in strict accordance with the recommendations in the Guide for the Care and Use of Laboratory Animals of AAALAC international. The protocol of human primary tumor establishment was reviewed and approved by Shanghai ChemPartner's IACUC (Permit Number: X998HL001). All HCC tumor tissues used for PDX model establishment were obtained from Nantong Tumor Hospital in accordance with protocols approved by the Institutional Ethics Committee of Nantong Tumor Hospital. All participants provided their written informed consent form. All tumor tissues were surgically resected and collected for the purposes of the study, and were appropriately de-identified except some information which was listed in Table 1. The HCC PDX model establishment at Shanghai ChemPartner Co., LTD was also reviewed and approved by independent Institutional Ethics Committee of School of Pharmacology, Central South University. All animal experiments were carried out following ChemPartner's IACUC guidelines in ChemPartner's animal facility which is fully accredited by AAALAC international. At the endpoint, the animals were euthanized by using CO_2_ and all efforts were made to minimize suffering.

**Table 1:** Information of patients.

Cell line	Age/sex	Clinical Pathological diagnosis	site	HBsAg	HCV	HIV
LIXC002	71/M	Poorly differentiated HCC	right lobe	+	−	−
LIXC003	49/M	Poorly differentiated HCC	middle lobe	+	−	−
LIXC004	39/F	Poorly differentiated HCC	left lobe	+	−	−
LIXC006	50/M	Poorly to moderately differentiated HCC	left lobe	+	−	−
LIXC011	36/M	Poorly differentiated HCC	right lobe	+	−	−
LIXC012	67/M	Poorly to moderately differentiated HCC	right lobe	−	−	−
CPL0903	51/M	Poorly differentiated HCC	right lobe	+	−	−

### Generation of PDX models

6–8 week-old female SCID mice (Beijing Vital River, China) were used for human HCC fragments implantation. Mice were maintained under specific-pathogen-free (SPF) conditions. The fresh tumor tissue specimens were rinsed twice with Hank's balanced salt solution (HBSS) containing antibiotic and transported on ice. As described previously[13], tumor tissues were cut into 2×2 mm pieces and implanted subcutaneously into mice. Tumor size and mice body weight were monitored up to 10 weeks and tumor volume (TV) was calculated by following formula: TV = 1/2×L×W^2^.

### Establishment of HCC cell lines from PDX models

The tumor-bearing mice were sacrificed and the tumors were removed when the tumors grew to 500-700 mm^3^. *In vitro* primary culture of HCC cells was initiated as described previously[13]. Briefly, the tumor tissues were minced and washed with culture medium, then transferred into a T-25 culture flasks and incubated at 37°C, 100% humidity with 5% CO_2_. The culture medium was RPMI 1640 medium supplemented with 10% fetal bovine serum (Invitrogen), 10 µg/ml human recombinant insulin and 1% Antibiotic-Antimycotic (Invitrogen, USA) containing amphotericin B, streptomycin and penicillin. Cells were subcultured at 70–80% confluence and used for further experiments after the passage 20. These cell lines were deposited at China Center for Typical Culture Collection (CCTCC). The access numbers could be found in Table S1 in File S1.

### Histomorphological and immunohistochemical staining

For morphology studies, cells were plated in 10-mm plates and grew to approximately 90% confluence. Phase images were obtained via inverted phase-contrast microscope (Olympus, Japan). For immunohistochemical staining, tumor cells were collected by centrifuging as cell pellet. Slowly added neutral formalin into the tube and fixed the cell pellet overnight. Then processed the cell pellet to paraffin block for embedding which could be used for making histological sections. After de-paraffinization and antigen retrieval procedure, cells were permeabilized with 0.1% Triton X-100, incubated with 0.3% H_2_O_2_ solution to quench endogenous peroxidase activity, and blocked with 4% goat serum (Invitrogen, USA) in PBS. The cells were incubated with the following primary antibodies at room temperature for 60 min: anti-AFP (Epitomics, USA. #7723-1), anti-CA19-9 (Santa Cruz, USA. Sc-59480), anti-CEA (Epitomics, USA. #5872-1), anti-ki-67 (Thermo, USA. # RM-9106), anti-Vimentin (Epitomics, USA. #2707-1) and anti-cytokeratin (Miltenyi Biotec, USA. #130-080-101). Anti-IgG1 was used as negative control. Then the slides were developed by DAB methods (Maxim, China) and mounted. The slides were observed and visualized by Eclipse Motorized Advanced Research Microscope (Nikon, Japan).

### Chromosome analysis

The chromosome specimens of the established HCC cell lines were valued as previously described[13]. Cells at logarithmic phase were harvested and suspended in 0.075 mol/L KCl hypotonic solution and then fixed in fix solution (methanol: glacial acetic acid = 3: 1). Chromosome specimens were stained with Giemsa and Chromosome numbers of M phase cells were counted under a microscope (Nikon, Japan). The chromosome frequency of each cell line was analyzed by Origin software.

### DNA isolation and STR (short tandem repeat) analysis

Genomic DNA from tumor cells was isolated using AxyPrep Multisource Genomic DNA Miniprep Kit (Axygen, USA) and 9 STR loci were analyzed by AmpF/STR® Identifiler® PCR Amplification Kit (ABI, USA).

### Growth kinetics *in vitro*

After establishment, growth kinetics of the tumor cells was obtained by seeding the cells at the density of 2000 cells/well into 96-well plates. The population doubling time was measured as previously described [13]. Briefly, the number of cells in each well was counted at a 12 h interval and the average value of triplicates was used to calculate the doubling time and plot their growth curve.

### Cell cycle

The cell cycle distribution was analyzed by flow cytometry. Cells (1×10^6^) were pre-seeded into T75 tissue culture flasks for 24 h. The harvested cells were washed with PBS and fixed with ice-cold 70% ethanol at −20°C overnight. The fixed cells were resuspended in PBS (containing 0.2 mg/mL RNAse and 10 µM PI) and incubated in the dark for 30 min at 37°C. A FACSCalibur flow cytometer (Becton Dickinson, San Jose, CA) was used for flow cytometric analysis.

### Quantitative RT-PCR

Total RNA from cultured cells was isolated using Trizol (Invitrogen, CA) according to the manufacturer's instructions. Reverse transcription was carried out with 0.5 µg of total RNA with PrimeScript™ RT Master Mix (Perfect Real Time) and oligo (dT) 15 primer (TAKARA Bio, China). Primer sequences used were obtained from the Primer-Bank database and sequences were provided in Table S2 in File S1. The reaction was carried out in a final volume of 20 µl with SYBR Green Premix Ex Taq (Takara Bio, China) on a Bio-Rad IQ5 Real-Time PCR System. A normal liver cell line HL 7702 was used as control.

### *In vitro* anti-proliferation assay

The cell populations were further characterized by analysing their reeponse to chemotherapeutic agents. Sorafenib (LC Lab, USA), docetaxel (LC Lab, USA), doxorubicin (Sigma), vinblastine (Sigma), tarceva (LC Lab, USA), gemcitabine (Chemiceutical, USA), oxaliplatin (Sigma) and cisplatin (Sigma) were added into triplicate wells at the same time of cell inoculation. A solvent control was performed at same time as the final DMSO concentration in medium was 0.5%. Cell viability was measured by CellTiter-Glo method (Promega, USA) after 96 h incubation and IC_50_ was determined by XLFit software.

### *In vivo* Tumorigenesis study

6–8 week-old female SCID mice were used for the xenograft tumor generation. As described previously [13], cells at different concentration were mixed with the matrigel (Becton Dickinson, US) at a ratio of 1: 1, and then inoculated subcutaneously into the right flank of the mice (10 mice for each line). The tumor growth and mice body weights were measured twice a week to plot the tumor growth curve. When the tumor grew to about 1500 mm^3^, the mice were euthanized and the tumors were collected. The viable tumor tissues were fixed in 4% formaldehyde, paraffin embedded and diagnosed (H&E staining).

### *In vivo* efficacy study

LIXC006 and LIXC012 cell lines were used to perform *in vivo* efficacy study. About 5.0×10^6^cells was injected into the mice to generate xenograft tumor. When the tumor grew to 100–250 mm^3^, twenty tumor-bearing mice were selected and divided into two groups. One group was treated with sorafenib 30 mg/kg intragastrically daily for 3 weeks. The other group was vehicle control group. The tumor growth was measured twice a week. At the end of test, tumor tissues were fixed in 4% formaldehyde, paraffin embedded and diagnosed (H&E staining).

### Statistical analysis

We used SPSS 18.0 software (IBM) for statistical analyses. Data were expressed as means ± SEM or S.D and were compared using one-way analysis of variance (ANOVA) followed by Student's *t* tests. Values of *p*<0.05 were considered to be statistically significant.

## Results

### Establishment and characterization of HCC cell lines

Human tumor tissues were obtained from surgical resection of Chinese patients. SCID mice were used to generate PDX models, and then primary culture of tumor cells was performed for cell lines establishment. We used 20 tumor tissues from different patients and got 7 PDX models, and then established 7 HCC cell lines which were designated as LIXC002, LIXC003, LIXC004, LIXC006, LIXC0011, LIXC012 and CPL0903. The information of patient related to cell line accordingly was listed in Table 1. They aged from 36 to 71 years old. One is female and the other six patients are male. 6 patients are HBV positive except one is negative. All patients are HCV and HIV negative.

After establishment, cell lines were banked at low passages and cells passaged at least 20 times were used for characterization. All cell lines were free of contamination by bacteria or mycoplasma. Their growth kinetics was shown in Table 2. The doubling time was ranged from 24.95 hours to 110.60 hours. All cells grew as monolayer and the cell morphology was captured by phase-contrast microscopy (data not shown). LIXC006 and LIXC012 cells grew with similar pattern which cells formed clusters. Cells of the other cell lines grew mimicking pavement in appearance.

**Table 2:** Growth kinetics of HCC cell lines.

Cell line	Growth kinetics				
	Pattern	G1 phase	S phase	G2/M phase	DT (h)
LIXC002	Monolayer	60.67±3.20%	20.64±3.90%	18.84±2.54%	44.71±0.38
LIX003	Monolayer	68.88±5.84%	15.24±1.30%	16.03±4.81%	24.95±0.43
LIXC004	Monolayer	54.48±6.36%	30.67±4.01%	14.86±2.23%	36.02±1.41
LIXC006	Monolayer	57.25±6.45%	27.40±5.29%	15.35±1.46%	52.82±0.93
LIXC011	Monolayer	54.70±0.93%	19.51±1.71%	25.80±2.64%	110.6±17.88
LIXC012	Monolayer	59.02±1.99%	27.76±7.60%	12.97±7.31%	76.68±4.16
CPL0903	Monolayer	57.38±7.36%	14.27±2.18%	28.06±9.03%	46.81±1.10

Data were expressed as the mean ± S.D. n = 3.

**Figure 1:**
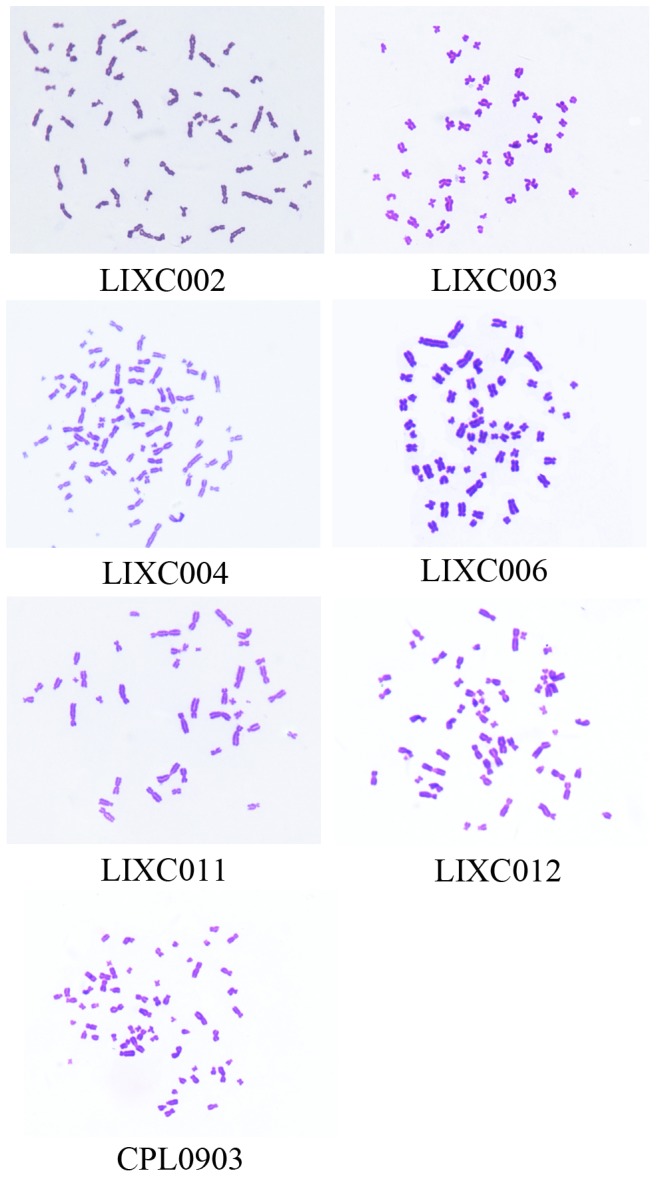
Chromosome analysis of 7 new HCC cell lines. The median centromere showed human cell origin. Magnification, ×1000.

Chromosome aberrations could be found in all cell lines. Most cell lines had more than 46 chromosomes except LIXC011 which had 35 chromosomes (Table.3). Fig. 1 also indicated the difference of chromosome and the human cell origin because of the median centromere. From STR assay, 8 tetranucleotide repeat loci (Table.4) and Amelogen gender determining markers were heterogeneously distributed in each cell line, and no cross-contamination was found in all cells. All these results suggested that the 7 cell lines were novel HCC cell lines.

**Table 3:** Quantification of chromosome aberrations.

Cell line	Percentage(%)	quantity
LIXC002	54.95	62±3
LIXC003	73.87	46±2
LIXC004	93.06	79±7
LIXC006	93.94	61±3
LIXC011	74.51	35±2
LIXC012	84.62	71±6
CPL0903	91.25	57±3

Data were expressed as the mean ± S.D. n = 3.

**Table 4:** DNA fingerprinting analysis using 9 STR loci for newly established 7 HCC cell lines.

STR Locus	LIXC002	LIXC003	LIXC004	LIXC006	LIXC011	LIXC012	CPL0903
Amelogenin	x, y	x, y	x	x, y	x, y	x, y	x, y
THO1	8, 9	9	7, 9	8	7	8	7, 9
TPOX	11	8, 9	8, 11	7	8, 11	7, 10	8, 11
D13S317	8, 14	10	11, 12	11	12	8, 12	8
vWA	14	14, 16	18, 20	13, 15	17	13, 17	16, 18
D16S539	11	11	9	9	11	8	11, 12
D5S818	12, 13	11	11	9, 11	13	11	11
CSF1PO	10, 13	9, 10	10, 11	9, 11	11	10, 11	10, 11
D7S820	11	12	8, 11	7, 10	8, 11	10, 11	12

### Detection of biomarkers and expression of mRNA level of some genes

**Figure 2:**
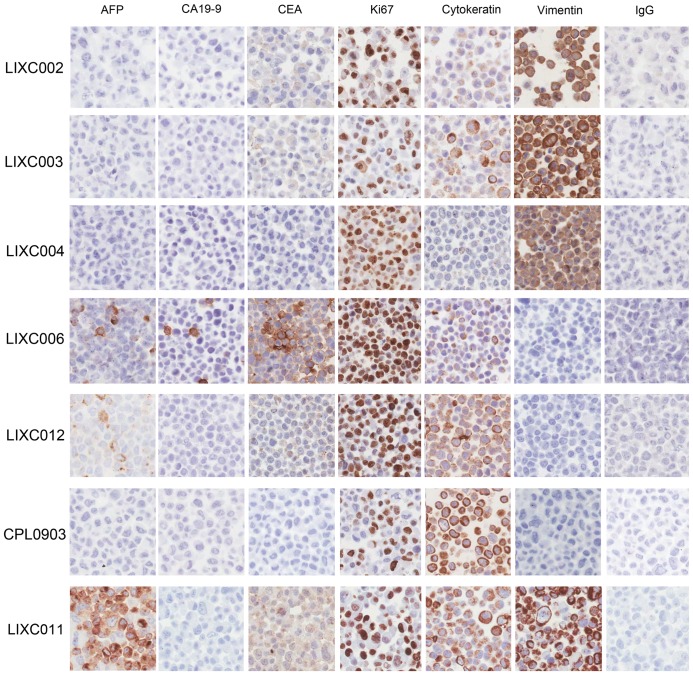
Immunohistochemical stain of the new HCC cell lines. 6 tumor related biomarkers were visualized by special antibodies using DAB methods in all 7 new HCC cell lines. IgG was negative control. Magnification, ×400.

The result of immunohistochemical staining for some biomarkers was shown in Fig. 2. AFP, a well-used biomarker for HCC[14], was positive in LIXC006, LIXC011 and LIXC012. Two well-known tumor markers for pancreatic cancer[15], carcinoembryonic antigen (CEA) and carbohydrate antigen 19-9 (CA19-9) were positive only in LIXC006. LIXC011 and LIXC012 were weakly stained for CEA. We also valued the concentration of AFP, CEA and CA19-9 in cell culture medium and the results (supporting information Table S3 in File S1) fitted well with the staining performance. Ki67, the protein which was associated with active cell proliferation, was found in nuclear (>10%) in all cell lines. Especially, LIXC004, LIXC006 and LIXC012 expressed very high level Ki67, indicating the poor prognosis of the donor patients[16]. Cytokeratin staining showed the epithelial phenotype of all 7 cell lines. Vimentin, the characteristic protein of mesenchymal cells, was expressed in LIXC002, LIXC004, LIXC006 and LIXC011, suggesting these HCC cells had high metastasis potential[17].

**Figure 3:**
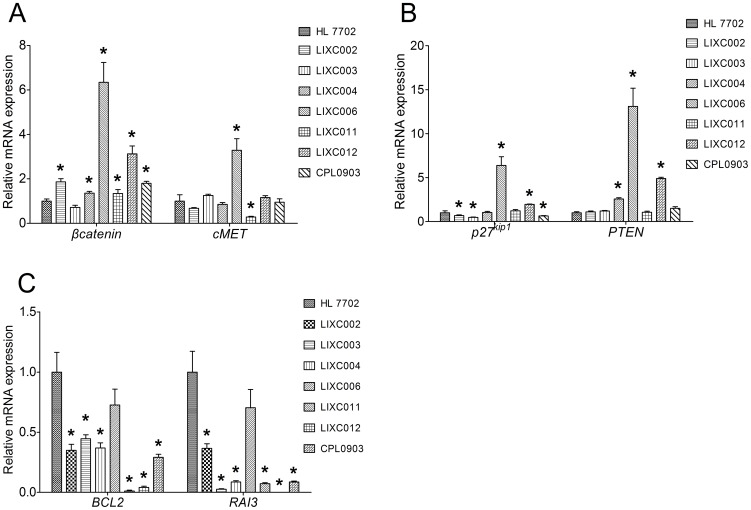
mRNA expression level of some tumor related genes in new cell lines. A, mRNA expression of *βcatenin* was up-regulated in all cell lines except LIXC003. LIXC006 cell expressed *cMET* mRNA significantly higher than HL 7702, while it was opposite in LIXC011. B, mRNA expression of *p27^kip1^* was down-regulated in LIXC002, LIXC003 and CPL0903 cell lines, but up-regulated in LIXC006 and LIXC012 cell lines. LIXC004, LIXC006 and LIXC012 expressed significantly higher *PTEN* mRNA than HL 7702. C, *BCL2* and *RAI3* mRNA was down-regulated in all cell lines. Data are expressed as the fold expression relative to the control HL 7702 cell and represented the mean ± S.D. of three individual experiments. *, *P*<0.05 compared with HL 7702 cell.

We also performed quantitative PCR to characterize mRNA expression level of some tumor related genes in the new cell lines. *βcatenin* and *cMet* are two proto-oncogenes. Overexpression of β-catenin is associated with many cancers including HCC[18]. As shown in Fig. 3.A, except LIXC003 cell, the other 6 cell lines expressed higher mRNA of *βcatenin* than normal HL 7702 cell (P<0.05). *cMet*, which encodes hepatocyte growth factor (HGF) receptor, was up-regulated in LIXC006, but down-regulated in LIXC011. HGF- cMET axis activation was implicated in cellular invasion and metastases[19]. P^27kip1^ was known as a protein of cyclin-dependent kinases inhibitor (CDKI)[20]. Its mRNA was down-regulated in in LIXC002, LIXC003 and CPL0903 cell lines, but overexpressed in LIXC006 and LIXC012 cell lines (Fig. 3.B). *PTEN*, which acts as a tumor suppressor gene through its phosphatase action, was often found lost its copies commonly in human cancers[21]. But in our study, LIXC004, LIXC006 and LIXC012 expressed significantly higher *PTEN* mRNA than HL 7702 (P<0.05) (Fig. 3.B). Bcl-2 is a well-known anti-apoptotic protein which regulates cell death. The mRNA level of *BCL2* gene was significantly decreased in all novel cell lines, indicating the increased proliferation potential of HCC (Fig. 3.C). Retinoic acid induced protein 3 (RAI3) is a member of G-protein-coupled receptors[22]. mRNA level of *RAI3* was decreased in all cell lines.

Our results demonstrated the diversity of these cell lines in biomarkers and tumor related genes.

### *In vitro* drug response

New cell lines were used for validation of anti-tumor drugs. 8 chemotherapeutic drugs, including sorafenib, docetaxel, doxorubicin, vinblastine, tarceva, gemcitabine, oxaliplatin and cisplatin, were tested for their anti-proliferation effect. IC_50_ results were demonstrated in Table 5. Except cell line CPL0903, the other 6 cell lines showed different sensitivity to the drugs. IC_50_ of sorafenib ranged from 0.93 to 13.57 µM in 6 cell lines. LIXC003 tolerated oxaliplatin but was sensitive to docetaxel. LIXC004 was resistant to docetaxel. LIXC12 was the most sensitive cell line among 7 cell lines to all drugs. We failed to detect IC_50_ in CPL0903 according to our dilute protocol. The cells might be clinical multiple drug resistant tumor cells. LIXC011 cell possessed moderately multiple drug resistance property with higher IC_50_ quantity for several drugs.

**Table 5:** *In vitro* anti-proliferation assay in HCC cell lines.

IC50 (μM)	Sorafenib	Oxaliplatin	Docetaxel	Gemcitabine	Vinblastine	Doxorubicin	Cisplatin	Tarceva
LIXC002	5.788	22.30	<0.032	0.018	0.004	0.688	10.725	13.601
LIXC003	11.971	>100	<0.032	0.050	0.009	0.895	18.465	22.890
LIXC004	7.077	30.22	>1	0.023	0.029	0.486	11.100	10.626
LIXC006	4.305	4.53	0.043	0.005	0.035	0.767	1.863	0.269
LIXC011	13.567	>100	>1	>20	28.36	2.732	29.405	17.577
LIXC012	0.929	7.50	<0.032	0.073	0.009	0.563	8.070	2.942

### *In vivo* tumorigenicity and drug response

**Figure 4:**
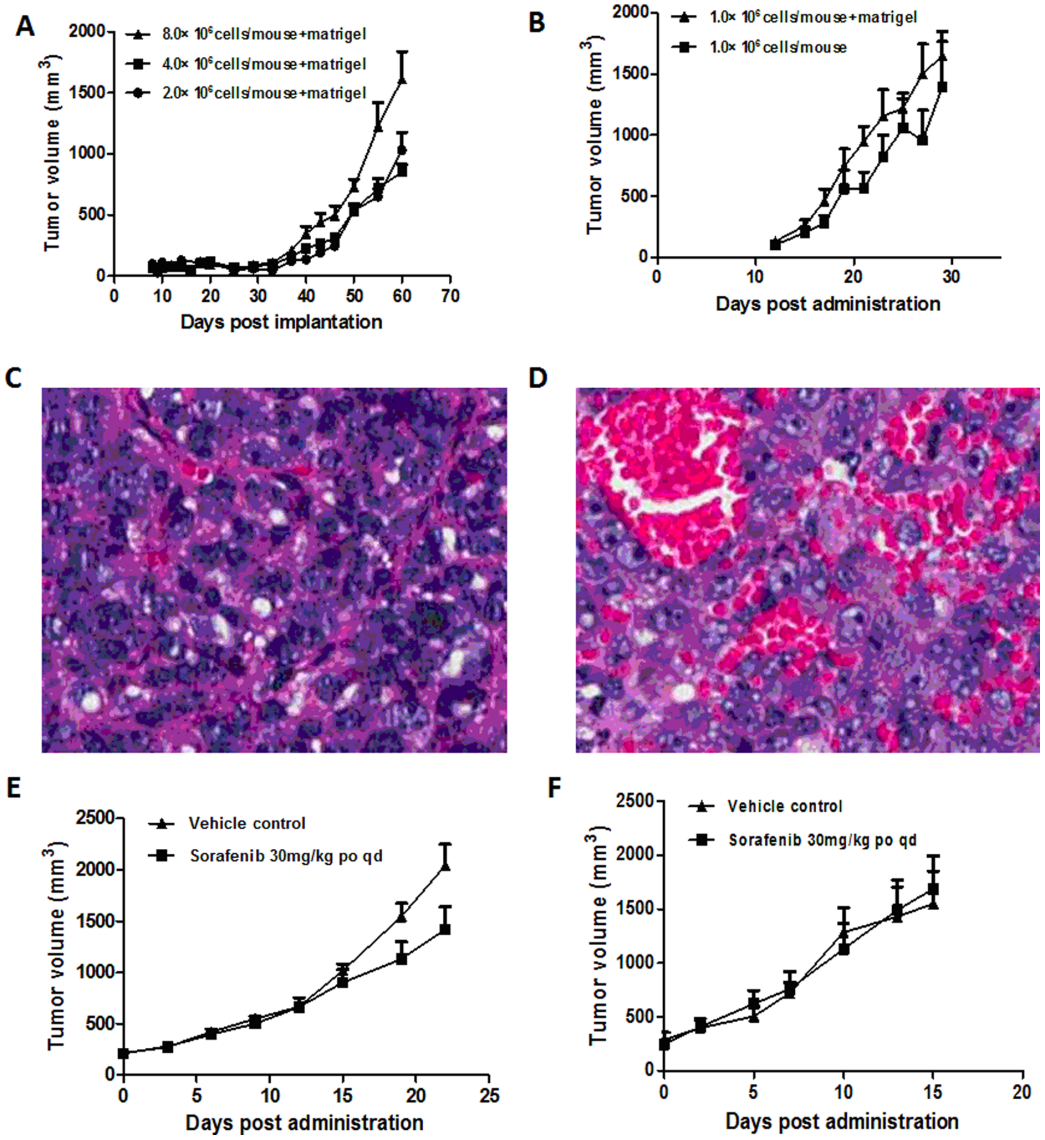
Tumor growth curve of LIXC006 and LIXC012 in tumorigenicity test, H&E staining of xenograft tumors and anti-tumor efficacy of sorafenib. Tumor volume was monitored twice a week. A, tumor growth curve of LIXC006 cells; B, tumor growth curve of LIXC012 cells. C, tumor tissue of LIXC006 cell, tumor cells were crowded together; D, tumor tissue of LIXC012, many blood vessels were inside tissue. Magnification, ×200. E, effect of sorafenib on xenograft tumor of LIXC006; F, effect of sorafenib on xenograft tumor of LIXC012. Data were presented as the mean ± SEM. n = 10.

*In vivo* tumorigenesis study was performed with all cell lines using SCID mice. Only LIXC006 and LIXC012 showed better tumorigenic ability. LIXC006 cell formed tumor with higher cell concentration and matrigel was necessary. LIXC012 cell formed xenograft tumor within 30 days, with lower cell concentration, no matter with or without matrigel. Tumor growth curve was plotted and presented in Fig. 4.A, B. When the tumor reached ∼1500 mm^3^, the tumor tissue was collected and H&E staining was performed, as shown in Fig. 4 C, D. Tumor tissue of LIXC006 was hard and cell grew crowded together, Fig. 4.C. On the other hand, tumor tissue of LIXC012 was soft and many blood vessels were inside tissue, Fig. 4.D. The abundant blood is accordance with the high speed of tumor growth. Result of *in vivo* drug reponse assay indicated that sorafenib showed anti-tumor efficacy in xenograft tumor of LIXC006 (Fig. 4.E). No significant effect was found in xenograft tumor of LIXC012 (Fig. 4.F). Tumor grew too fast to be inhibited.

## Discussion

China has high incidence of HCC because of the highest prevalence of HBV infection in the world[23]. Chronic HBV infection is one of the most serious infectious diseases in China and brings big burden for society. In this study, we reported the establishment of 7 new HCC cell lines from PDX models which the tumor samples came from Chinese patients. Histomorphological, genetical, biochemical, and molecular biological assays were performed to characterize the new HCC cell lines. *In vitro* anti-proliferation assay with all these cell lines indicated the different sensitivities to 8 well used chemotherapeutic drugs. Two cell lines with better tumorigenic ability were used for validation the anti-tumor efficacy of sorafenib *in vivo*. Our results suggested that the 7 new HCC cell lines with high diversity, could be used not only to study the molecular pathogenesis but also to investigate possible etiological agents of human HCC.

Except LIXC012 cell, the other 6 cell lines originated from HCC patients with HBV infection. All these new cell lines have different characters. Genetical study indicated that LIXC003 cell line likely had normal 46 chromosomes. Most other cell lines had more than 46 chromosomes whereas LIXC011 had 35 chromosomes. LIXC003 cells grew very fast with doubling time of 24.95 h, while LIXC011 cells' doubling time was as long as 110.6 h. LIXC012 did not grow fast *in vitro* and the doubling time was 76.68 h. But *in vivo*, LIXC012 formed xenograft tumor so fast that sorafenib failed to inhibit the tumor growth. Our results demonstrated the diversity of these new cell lines which was in accordance with the heterogeneity of HCC cells.

DNA fingerprinting is a valuable approach to authenticating and characterizing cell lines[24]. DNA fingerprinting analysis should be conducted to rule out the mycoplasma and other bacteria contamination when new cell lines are being established. Our study demonstrated unique profiles for the 7 cell lines without any microorganism contamination. These cells lines can be used for further study.

AFP is secreted in approximately 70% of HCC. Measurement of AFP has been used to diagnose HCC and monitor the response to HCC therapy[25]–[27]. Some recent studies showed determination lacks adequate sensitivity and specificity for effective surveillance and diagnosis[28], [29]. In our study, 3 out of 7 cell lines secreted abnormal amount of AFP. Combination of AFP and other molecule such as des-γ-carboxyprothrombin can increase the sensitivity in early-stage patients[29], [30]. Positive immunostain nucler Ki67 expression (>10%) was found in all new cell lines. Combining with the clinical pathological diagnosis, these HCC had high recurrence potential and poor prognosis. We also noticed that vimentin, the marker protein for epithelial-to-mesenchymal transition (EMT)[31], was atypically overexpressed in 4 cell lines. Hu *et al* demostrated that the abnormal expression of vimentin in HCC associated with local invasiveness and metastasis potential[17]. So, defination of sensitive and specific markers for early dignosis is much more important. The novel HCC cell lines with low-passage provided a useful tool for future investigations.

We characterized the mRNA expression profile of some tumor related genes including proto-oncogenes and tumor suppressor genes. Unexpectedly, 3 cell lines overexpressed *PTEN* aberrantly. Further study should be carried out to value the mutations and its enzymatic activity. Retinoic acid (RA) and its homologs, which function via nuclear RA receptors (RARs), are potent regulators of gene expression. Compounds that target these receptors are powerful anticancer drugs[32], [33]. Abnormal expression and function of RARs are often involved in the growth and development of cancer[34], [35]. Low serum retinol levels correlates with HCC risk[36]. Retinoic acid induced 16 (RAI16) was proved to be a useful therapeutic agent for HCC gene therapy and tumor marker for diagnosis. We explored another RA induced protein RAI3 and found its mRNA expression was significantly down-regulated in all new cell lines. It still need confirmation through adequately designed studies to make sure whether it could be another therapeutic target or tumor marker for HCC diagnosis.

Cell systems as tools and models for basic clinical research have been progressively increased during last few decades. We performed drug validation using the new HCC cell lines and got very interesting results. 8 chemotherapeutic drugs, including doxorubicin, gemcitabine, sorafenib and so on, were validated in our study. The results were quite individual. LIXC006 and LIXC012 were more sensitive to all drugs among the 7 cell lines. Some cell lines, such as LIXC011, showed multiple drug resistance potential towards all tested drugs. Sorafenib, a multi-kinase inhibitor which has been approved worldwide as a new target agent for HCC[37], [38], was effective in all cell lines *in vitro*, so was in mice bearing xenograft tumor of LIXC006. LIXC012 cell, the most sensitive to sorafenib with 0.929 µM as IC_50_, lost its sensitivity to the drug *in vivo*. It indicated that results based on *in vitro* should be carefully validated. Plenty of work could be carried out in future to figure out the relevance between cell molecular profile and drug sensitivity.

The interest of PDX models in preclinical research is more and more emphasized. Since it could reproduce the tumor microenvironment and tumor cell interactions with the innate immune system, PDX models offer a powerful tool for studying tumor biology and for evaluating anticancer drugs[11]. Researchers used PDX models to represent tumor heterogeneity to evaluate novel targeted therapeutic strategies[39], [40]. HCC is a highly heterogeneous disease and the clinical outcome is very variable between individuals[7], [10]. There is no effective treatment options for advanced disease. In addition, even in the case of patients with successfully curative therapies, the recurrence rate of HCC is as high as 50% at 2 years. The molecular mechanisms of this cancer are not well understood, therefore, we still need good model systems to study HCC. The new established cell lines with low-passage can be used to identify molecular features that predict or indicate response to targeted therapies.

## Supporting Information

File S1 **Contains the files:** Table S1. The access number of novel cell lines in China Center for Typical Culture Collection (CCTCC). Table S2. The primer sequences. Method: Electrochemiluminescence immunoassay (ECLI). Table S3. Concentration of AFP, CEA and CA19-9 in culture medium.
(DOCX)
