# Supplementary material for: Establishment and Characterization of 7 Novel Hepatocellular Carcinoma Cell Lines from Patient-Derived Tumor Xenografts
Source: PLoS One. 2014 Jan 9;9(1):e85308. doi: 10.1371/journal.pone.0085308 (PMC3887059; doi:10.1371/journal.pone.0085308)
Supplement: File S1 — Contains the files: Table S1. The access number of novel cell lines in China Center for Typical Culture Collection (CCTCC). Table S2. The primer sequences. Method: Electrochemiluminescence immunoassay (ECLI). Table S3. Concentration of AFP, CEA and CA19-9 in culture medium. (DOCX) [file pone.0085308.s001.docx]

**S 1**

**Table S1. The access number of novel cell lines in China Center for Typical Culture Collection (CCTCC).**

| Cell line | CCTCC number |
| --- | --- |
| LIXC002 | C201072 |
| LIXC003 | C201112 |
| LIXC004 | C201073 |
| LIXC006 | C2013182 |
| LIXC011 | on going |
| LIXC012 | C201114 |
| CPL0903 | on going |

**Table S2. The primer sequences.**

| Gene | PrimerBank ID | Forward | Reverse |
| --- | --- | --- | --- |
| *βcadherin* | 148233337c3 | AGCTTCCAGACACGCTATCAT | CGGTACAACGAGCTGTTTCTAC |
| *p27^Kip1^* | 207113192c2 | ATCACAAACCCCTAGAGGGCA | GGGTCTGTAGTAGAACTCGGG |
| *MET* | 188595715c2 | AGCGTCAACAGAGGGACCT | GCAGTGAACCTCCGACTGTATG |
| *PTEN* | 110224474c3 | AGGGACGAACTGGTGTAATGA | CTGGTCCTTACTTCCCCATAGAA |
| *RAI3* | 63252917c1 | ATGGCTACAACAGTCCCTGAT | CCACCGTTTCTAGGACGATGC |
| *BCL2* | 6456033a2 | CCAGCGTATATCGGAATGTGG | CCATGTGATACCTGCTGAGAAG |
| *βACTIN* | 144922730c1 | GTCTGCCTTGGTAGTGGATAATG | TCGAGGACGCCCTATCATGG |

**Method: Electrochemiluminescence immunoassay (ECLI)**

Cells (1x10^6^) were seeded into T75 tissue culture flasks and cultured for 24 h. The supernatant of culture medium was collected by centrifuging and the cells were used for RNA isolation. ECLI was performed at Roche Cobas E601 automatic immune analyzer with Elecsys AFP, Elecsys CEA and Elecsys AFP, which were all purchased from Roche, USA. Experiments were performed in triplicate, and data represent the mean of three individual experiments.

**Table S3. Concentration of AFP, CEA and CA19-9 in culture medium.**

| Cell line | AFP ng/ml | CEA ng/ml | CA19-9 ng/ml |
| --- | --- | --- | --- |
| HL 7702 | 0.605 | 0.2 | 2.36 |
| LZXC002 | 0.781 | 0.2 | 2.7 |
| LZXC003 | 0.605 | 0.2 | 3.54 |
| LZXC004 | 0.605 | 0.2 | 2.48 |
| LZXC006 | 312.3 | 2.98 | 3.81 |
| LZXC011 | 157.8 | 0.2 | 2.53 |
| LZXC012 | 285.5 | 0.2 | 3.62 |
| CPL0903 | 0.605 | 0.2 | 3.71 |
